# The Influence of the External Signal Modulation Waveform and Frequency on the Performance of a Photonic Forced Oscillator

**DOI:** 10.3390/ma11050854

**Published:** 2018-05-21

**Authors:** Noemi Sánchez-Castro, Martha Alicia Palomino-Ovando, Denise Estrada-Wiese, Nydia Xcaret Valladares, Jesus Antonio del Río, Maria Beatriz de la Mora, Rafael Doti, Jocelyn Faubert, Jesus Eduardo Lugo

**Affiliations:** 1 Faculty of Physics and Mathematics, BUAP, Avenida San Claudio y 18 Sur, Colonia San Manuel, Edificio FM1, Ciudad Universitaria, Puebla 72570, Mexico; sc.noemi@gmail.com (N.S.-C.); marthap@fcfm.buap.mx (M.A.P.-O.); 2 Faubert Lab, School of Optometry, University of Montreal, Montreal, QC H3C3J7, Canada; rafael.doti@gmail.com (R.D.); jocelyn.faubert@gmail.com (J.F.); 3 Instituto de Investigación en Ciencias Básicas y Aplicadas, Universidad Autónoma del Estado de Morelos, Avenida Universidad No. 1001 Col. Chamilpa, Cuernavaca, Morelos 62209, Mexico; de.e.wiese@gmail.com; 4 Instituto de Energías Renovables, Universidad Nacional Autonóma de México, Privada Xochicalco S/N, Temixco, Morelos 62580, Mexico; nxvaa@ier.unam.mx (N.X.V.); arp@ier.unam.mx (J.A.d.R.); 5 CONACyT Fellow-CCADET Universidad Nacional Autónoma de México (UNAM), Ciudad de México 04510, Mexico; betarina@gmail.com

**Keywords:** photonic oscillator, photodyne, photonic crystal, electromagnetic forces

## Abstract

Photonic crystals have been an object of interest because of their properties to inhibit certain wavelengths and allow the transmission of others. Using these properties, we designed a photonic structure known as photodyne formed by two porous silicon one-dimensional photonic crystals with an air defect between them. When the photodyne is illuminated with appropriate light, it allows us to generate electromagnetic forces within the structure that can be maximized if the light becomes localized inside the defect region. These electromagnetic forces allow the microcavity to oscillate mechanically. In the experiment, a chopper was driven by a signal generator to modulate the laser light that was used. The driven frequency and the signal modulation waveform (rectangular, sinusoidal or triangular) were changed with the idea to find optimal conditions for the structure to oscillate. The microcavity displacement amplitude, velocity amplitude and Fourier spectrum of the latter and its frequency were measured by means of a vibrometer. The mechanical oscillations are modeled and compared with the experimental results and show good agreement. For external frequency values of 5 Hz and 10 Hz, the best option was a sinusoidal waveform, which gave higher photodyne displacements and velocity amplitudes. Nonetheless, for an external frequency of 15 Hz, the best option was the rectangular waveform.

## 1. Introduction

The use of light concepts such as radiation pressure and optical tweezers to spatially manipulate micro-objects or to levitate viruses, bacteria, cells, and subcellular organisms have been around since the 1970s [1,2]. Currently, there is a fast development of electromagnetic wave driven micro motors [3] where the radiation pressure is too small to activate such devices [4]. However, some resonance principles can be used to notably increase the force, for instance in waveguides made of lossless dielectric blocks or Bragg waveguides [5,6,7]. Due to the vast range of optical properties that can be obtained by the design of a photonic crystal, the creation of devices derived from the application of these properties has increased [8,9,10,11]. An interesting application using photonic crystals is the confinement of an incident electric field that allows the emergence of electromagnetic forces on the surface of the layers inside the photonic crystal [4,12]. Recently, we realized such application using one-dimensional photonic crystals (1D-PC) based on Porous Silicon (Psi) multilayers to create a dynamical system capable of performing oscillations such as a forced oscillation. In this application, an oscillation is imposed upon the system by an external source (vibrator) at a certain frequency [13,14,15]. The photonic crystal structures known as photodynes were activated with modulated laser light. Light modulation was achieved with a mechanical chopper and a wave generator. Square waveforms were used at different duty cycles and light intensities. Under these conditions, the photonic structure, amplitude and frequency were measured. From these measurements and with the help of a theoretical model, a pendulum in a viscous frictional medium acted upon by a force of constant magnitude, the induced electromagnetic force was estimated. The obtained values are of the order of a few nN to hundredths of nN. Notwithstanding these remarkable results, we would like to emphasize that we did not explore the influence of the external signal modulation waveform and frequency on the performance (amplitude and oscillation frequency) of the photonic oscillators. 

In this paper, the experimental details for the fabrication of photonic crystals and its characterization are described and the measurement of the photonic crystal oscillations is presented. Then, the theoretical model that describes the oscillations is analyzed. Subsequently, the comparison between the theoretical analysis and the experimental results is shown. Finally, some conclusions are given.

## 2. Materials and Methods

### 2.1. Porous Silicon Fabrication

Psi was fabricated by wet electrochemical anodization of highly boron-doped c-Si substrates with orientation (100) and electrical resistivity of 0.001–0.005 Ohm–cm (room temperature = 25 °C, humidity = 30%). On one side of the c-Si wafer, an aluminum film was deposited and then heated at 550 °C for 15 min in a nitrogen atmosphere to produce a good electrical contact. To have flat interfaces, an aqueous electrolyte composed of HF/ethanol/glycerol was used to anodize the silicon substrate. It is well known that the Psi-refractive index increases by decreasing the electrical current applied during the electrochemical etching. To produce the multilayers, current density applied during the electrochemical dissolution was alternated from $3\;\mathrm{mA}/{\mathrm{cm}}^{2}$ (layer 1) to $40\;\mathrm{mA}/{\mathrm{cm}}^{2}$ (layer 2) and 15 periods (30 layers) were made. 

### 2.2. Photonic Bandgap Structure

**Figure 1:**
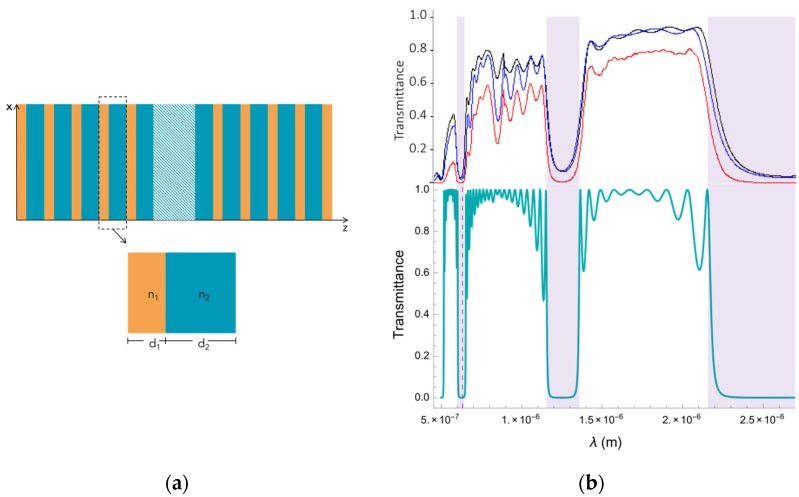
(**a**) Photonic device used in the experiment. Yellow and blue layers represent the Psi multilayer, while light blue layer is the air defect in the middle of the structure. (**b**) The measured transmittance of the complete multilayered device of porous silicon (top) has been compared with the theoretical model (bottom). The band gaps coincide in all cases, specifically in the selected working region (around 633 nm), for the refractive index of $n_{1}=1.1$ and $n_{2}=2$ and thicknesses of $d_{1}=335\;\mathrm{nm}$ and $d_{2}=438\;\mathrm{nm}$. The transmittance line of the multilayer used in the experiment is the black line.

The photonic bandgap structure was determined by measuring the transmittance spectrum of the structures, which is shown in Figure 1a. The well-known Transfer Matrix Method was used to fit the experimental spectrum. We designed the photonic crystals to work at a wavelength of 633 nm which lies within the third photonic bandgap. The best refractive indices and thickness values we found that fit the experimental photonic bandgap structure are $n_{1}=1.1$, $n_{2}=2$ and $d_{1}=335\;\mathrm{nm}$, $d_{2}=438\;\mathrm{nm}$. 

### 2.3. Photodyne Fabrication

**Figure 2:**
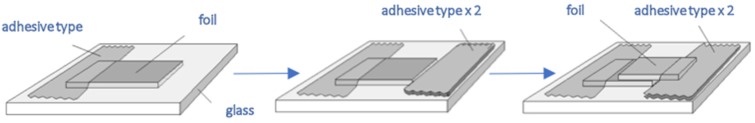
Configuration of the photodyne structures.

Photodyne structures were fabricated in a double juxtaposed cantilever configuration (see Figure 2). First, a Psi multilayer foil was placed over a flat glass substrate. Second, to place a second Psi multilayer foil, a spacer was used to compensate for the thickness due to the already placed Psi multilayer foil. Finally, the second Psi foil multilayer was placed on top of the first foil to form the final structure. Thus, there were two Psi 1D-PCs in a mirror-like symmetry with a gap space between them. Due to the type of configuration (known also as a microcavity), the separation between both mirrors cannot be controlled. It was also observed that both movable parts of the photodyne have an irregular surface, that is, the length of the air layer (the length of the microcavity) was different for each area on this surface. This could be called the *dangling defect condition*. In previous works [13,14], the length of the defect layer (air) was measured with a CCD camera with an optical objective (focal length 8 mm, 5 microns resolution) that goes from 5 μm up to 1 mm. The electromagnetic force would exist but would not have the maximum value when the defect conditions are not reached. In the same references, the electromagnetic force, under the aforementioned conditions, was calculated and ranged from $2\;\mathrm{mN}/m^{2}$ to $3.5\;\mathrm{mN}/m^{2}$. Therefore, the frequencies that can be coupled within this cavity were also dynamic and depended on the microcavity length changes. 

### 2.4. Experimental Setup

**Figure 3:**
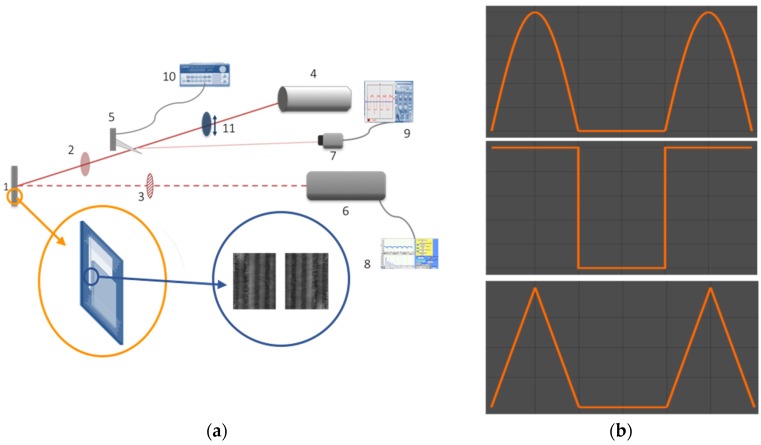
(**a**) Experimental setup: Photodyne (1), Neutral filter wheel (2), infrared band-pass (3), He-Ne Laser (4), mechanical chopper (5), vibrometer (6), photocell (7), computer (8), oscilloscope (9), function generator (10), and lineal polarizer (11). The drawing in the orange circle shows the mounted photodyne. A picture of the multilayers produced by SEM can be seen in the blue circle. (**b**) An example of ideal waveforms of the signals used in the experiments. From top to bottom: sinusoidal, rectangular and triangular.

The photodyne device was mounted on a rotary and XY linear stage (a representative drawing is shown in Figure 3a). A function generator controled the movement of a chopper that blocks or allows the red laser light (633 nm) to pass according to the signal sent by the function generator. The light power was 13 mW with an angle of incidence of 35 degrees and TE polarization. The size of the laser spot was approximately $3{\;\mathrm{mm}}^{2}$. The waveforms we used for the signals were sinusoidal, triangular and rectangular. The frequency of the signals was also a parameter to be tested. Frequencies of 5 Hz, 10 Hz and 15 Hz were used. A drawing showing ideal waveforms of the signals can be seen in Figure 3b. The offset signal was chosen to have a duty cycle of approximately 40%. The induced mechanical movement in the photonic crystal was measured by the high precision vibrometer (metro laser, model 500 v). By using an oscilloscope and a photocell, the signal was verified, and desired pulses of ±5 VDV and the presence of a 60 Hz line noise signal were recognized. To prevent undesirable reflection signals entering the vibrometer, an infrared band-pass filter was used. The option of playing with different power light intensities was considered as well as adding a neutral wheel filter. The diagram of the experiment is presented in Figure 3a. Considering that circuit, when the chopper allows for the energy provided by the polarized laser light to pass, the electromagnetic force push downs the upper part of the photodyne, and the system starts an oscillatory movement with period T.

### 2.5. Oscillations Theoretical Model

In this section, a short summary is made about the emergence of electromagnetic forces that make the photodyne oscillate. Then, the nature of the parameters and the oscillations in the device are analyzed. Consider light impinging on the off-axis direction at angle *θ*_0_ with the electric field polarized in the *y*-direction with a magnitude of:

(1) $$E_{y}=E\left(x\right)e^{i\left(\omega t-\beta z\right)},$$

where

(2) $$E\left(x\right)=\{\begin{array}{c} A_{0}e^{-jk_{0}\left(x-x_{0}\right)}+B_{0}e^{jk_{0}\left(x-x_{0}\right)}\;\;\;\;x<x_{0}\\ A_{l}e^{-jk_{1}\left(x-x_{1}\right)}+B_{l}e^{jk_{1}\left(x-x_{1}\right)}\;\;x_{l-1}<x<x_{1}\\ \;\;\;\;A_{s}e^{-jk_{s}\left(x-x_{N}\right)}+B_{s}e^{jk_{s}\left(x-x_{N}\right)}\;\;\;\;x_{N}<x \end{array}.$$

In Equation (2) *A_i_* and *B_i_* are the complex amplitudes of the electric field in each region of the structure. The 0 label is for the amplitudes in the air region. The wave vectors are represented by *k_i_* at different regions on the structure in the *x*-direction. *β* is the wave vector in the *z*-direction and is given by $\omega n_{0}\sin\left(\theta_{0}/c\right)$, where $n_{0}$ and $\theta_{0}$ are the refractive index and angle of incidence of the air region, c is the speed of light and ω is the light angular frequency. The wave vectors in the *x*-direction are given by $\omega n_{0}\cos\left(\theta_{i}/c\right)$, where $n_{i}$ and $\theta_{i}$ are the refractive index and angle of incidence of region I, the latter given by $\theta_{i}=\sin^{-1}(n_{0}\sin\theta_{0}/n_{i})$. By using a similar formalism as presented in [9,10,11,12,13,14,15,16], it can be shown that for lossless dielectrics the surface force density only exists in the *x*-direction and is given by:

(3) $$\begin{array}{l} \langle F_{x}\rangle {}_{T}=\overset{N}{\underset{l=1}{\sum }}\frac{\varepsilon_{0}}{4}\left[{\left(\frac{n_{l}-1}{n_{l}}\right)}^{4}-1\right]\left[{\left|A_{l}\right|}^{2}+{\left|B_{l}\right|}^{2}+2\left|A_{l}\right|\left|B_{l}\right|\cos\left(2k_{1}d_{1}+\varphi_{A_{l}}-\varphi_{B_{l}}\right)\right]\\ \;+\frac{\varepsilon_{0}}{4}\left[{\left(\frac{n_{N}}{n_{s}}\right)}^{4}-1\right]\left[{\left|A_{s}\right|}^{2}+{\left|B_{s}\right|}^{2}+2\left|A_{s}\right|\left|B_{s}\right|\cos\left(\varphi_{A_{s}}-\varphi_{B_{s}}\right)\right]\\ \;+\overset{N}{\underset{l=1}{\sum }}\frac{\varepsilon_{0}}{2}\left[{\left(n_{l}\right)}^{2}-1\right]\left|A_{l}\right|\left|B_{l}\right|\left[\cos\left(\varphi_{A_{l}}-\varphi_{B_{l}}\right)-\cos\left(2k_{l}d_{l}+\varphi_{A_{l}}-\varphi_{B_{l}}\right)\right], \end{array}$$

where $\varepsilon_{0}$ is the vacuum permittivity and *N* is the total number of layers. The complex amplitudes *A_i_* and *B_i_* and their phases *φ_i_* can be calculated by using the well-known transfer matrix method [2]. 

Using the magnitude of the force, the acceleration of the movement originated due to these forces can be calculated. The movement equations of a simple oscillating system are used to begin the study. A pendulum in a viscous frictional medium acted upon by a force of constant magnitude is a dynamical system that can be modeled by the next differential equations:

(4) $$\begin{array}{l} \ddot{x}+2h\dot{x}+\omega_{0}^{2}x=f\left(t\right)\langle a_{x}\rangle {}_{T}\;jT<t<\left(n_{light}+j\right)T\\ \ddot{x}+2h\dot{x}+\omega_{0}^{2}x=0\;\left(n_{light}+j\right)T<t<\left(j+1\right)T\\ \;j=0,\ldots ,m \end{array}$$

where *h* is the damping coefficient, $\omega_{0}$ is the natural frequency of the system, $n_{light}$ defines the duty cycle (fraction of the period where the light is on) and j+1 is the number of cycles that the light is off and on. The period *T* is given by 2π/ω, where ω is the external driving frequency. In this system, the driving force of the forced oscillator is the electromagnetic force per mass unit $\langle a_{x}\rangle {}_{T}$ = $\langle F_{x}\rangle {}_{T}A/m_{psi}$, where $m_{psi}$ and A are the mass and the active surface area of the Psi photodyne. The term $2h\dot{x}$ is proportional to the viscous frictional force and $\omega_{0}^{2}x$ is proportional to the harmonic force. The variable *x* is the amplitude of the movement. Therefore, $\dot{x}$ and $\ddot{x}$ constitutes the velocity and acceleration, respectively. When the chopper cuts the light off, there will be no driving force, which is mathematically represented by the duty cycle. To study the amplitude behavior of the photodyne oscillations, three different signal forms were tested (sinusoidal, rectangular and triangular). We introduced these three waveforms into the model through function f(t). For the triangular wave form:

$$f\left(t\right)=\{\begin{array}{c} \left(20/\pi \right)\omega t-1\;\;\;\;\;jT<t<\left(j+1/20\right)T\\ \left(-20/19\pi \right)\omega t+21/19\;\;\;\left(j+1/20\right)T<t<\left(j+1\right)T \end{array}$$

For the sinusoidal waveform:

f(t)=sinωt   jT<t<(j+1)T

and for the rectangular signal: f(t)=1  jT<t<(j+1)T. *j* is the same number as before. It should be noted that the final driving signal waveform for the incident light in the photodyne is the convolution of a rectangular function given in Equation (4) and the function f(t). 

The damping coefficient and the induced electromagnetic force per mass unit were taken as free parameters. Although the experimental duty cycle was fixed we found better theoretical results if the duty cycle was a free parameter within the range of 40%±10%. 

As reported in previous work [13,14], for the exact same photodyne configuration, an auto-oscillating system was used for the calculation of the value $\omega_{0}$, which equals 153.9 rad/s. The amplitude of the oscillations depends on the form of these signals, however, as mentioned before, due to the configuration of the photodyne, they are also subjected to the initial conditions of the system (initial size of the cavity and uniformity of the surface) and the duty cycle. These factors can have an impact on the final oscillation amplitude and the model partly captures these effects through the duty cycle and damping coefficients. However, other nonlinearities due to the initial cavity size and surface uniformity are more difficult to introduce theoretically. 

Theoretically, we expect a direct relation between the number and amplitude of the oscillations with the duty cycle of the light *n_light_* for any waveform signal used here, as well as an inverse relation between the amplitude of oscillations and the damping coefficient *h*. The electromagnetic force per mass unit parameter controls the total energy provide to the photodyne. This energy controls the maximum amplitude of the oscillations. Therefore, for any waveform, the oscillation amplitude depends on the combination of these parameter values. That is, the maximum theoretical mechanical power used by the photodyne is given by the combinations of these three parameters.

## 3. Results

**Figure 4:**
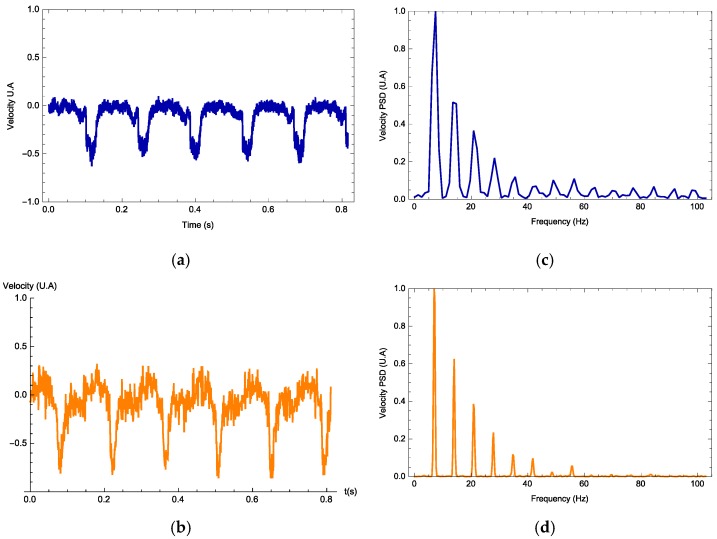
Photodyne movement velocity (**a**,**b**) and Fourier spectrum (**c**,**d**). Comparison of the theoretical (orange) and experimental results (blue). Rectangle signal, input frequency of 5 Hz. Calculated value of *h* = 41.4 and *n_ligth_* = 0.42.

**Figure 5:**
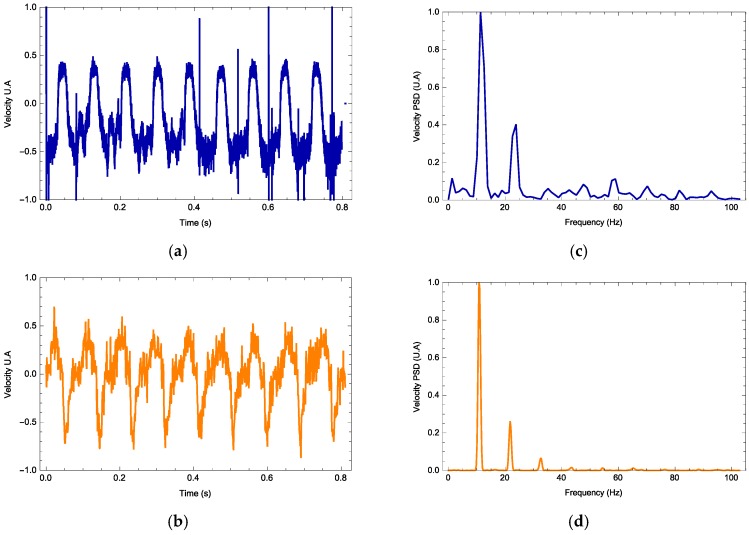
Photodyne movement velocity (**a**,**b**) and Fourier spectrum (**c**,**d**). Comparison of the theoretical (orange) and experimental results (blue). Sinusoidal signal, input frequency of 10 Hz. Calculated value of *h* = 27.1 and *n_ligth_* = 0.50.

**Figure 6:**
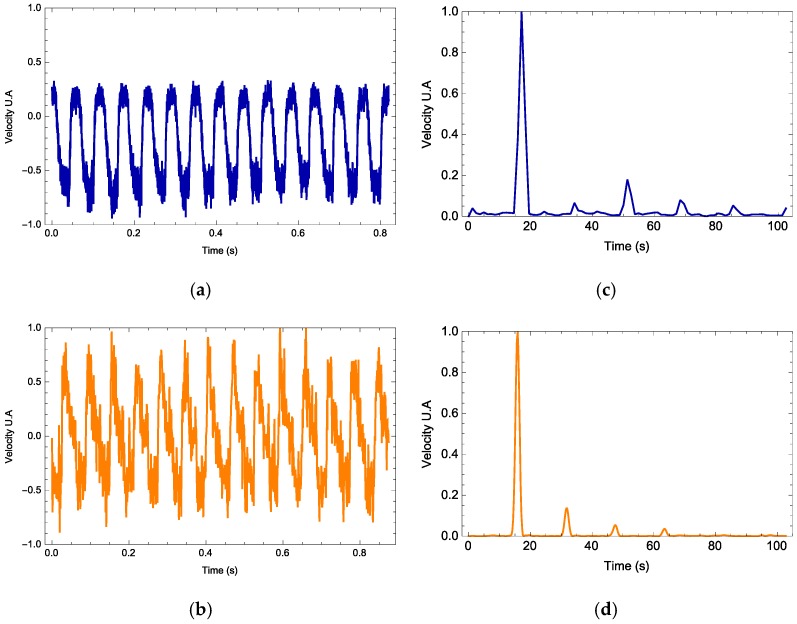
Photodyne movement velocity (**a**,**b**) and Fourier spectrum **(c**,**d**). Comparison of the theoretical (orange) and experimental results (blue). Rectangle signal, input frequency of 15 Hz. Calculated value of *h* = 33.2 and *n_ligth_* = 0.5.

The vibrometer software interface provides the data containing the maximum displacement and velocity, velocity time series and its Fourier spectrum along with its mean frequency and harmonics. In all experiments, the vibrometer gives the velocity profile as a signal measured in volts. For each signal waveform, the parameters *h*, $n_{light}$, and $\langle a_{x}\rangle {}_{T}$ are free parameters and Equation (1) is calculated. In Figure 4, Figure 5 and Figure 6, we observe three examples (one for each waveform) on how the photodyne velocity oscillates. Both theory and experimental results are compared. In general, the theory can predict correctly the velocity waveform and the number of harmonics present on the Fourier Power Spectral Density. In all examples, the best value for $\langle a_{x}\rangle {}_{T}$ was 1.98 m/s^2^ while *h* and $n_{light}$ took different values in each example.

Based on the experimental data and the theoretical model, it is possible to know for each frequency which type of signal is the best to optimize the displacement of the photodyne. 

**Figure 7:**
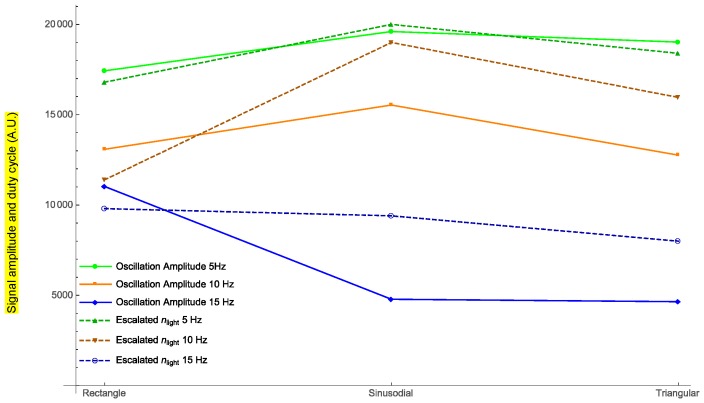
Behavior of the photodyne displacement (in nanometers) against three different types of waveforms. The parameter *n_light_* has been normalized for the representation.

In Figure 7, the amplitude of the oscillations for three different external frequencies is plotted for each signal waveform. Dashed lines show the duty cycle of the light obtained for each case (*n_light_* has been normalized for the representation).

**Figure 8:**
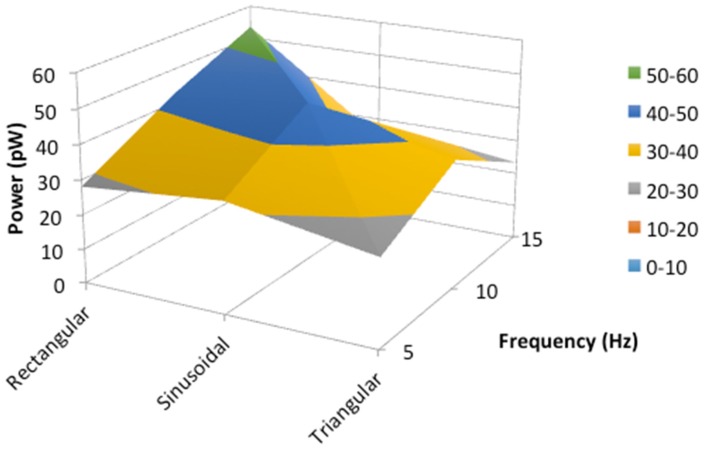
Maximum power of the oscillation movement against the three external frequencies and waveforms.

The maximum mechanical power of the oscillation movement has been calculated by multiplying the maximum experimental velocity times the calculated electromagnetic force. In Figure 8, the maximum mechanical power of the oscillations for three different external frequencies are plotted for each signal waveform.

## 4. Discussion and Conclusions

Electromagnetic forces due to an incident electromagnetic field are induced within a photonic device known as a photodyne. Photodynes are based on one-dimensional photonic crystals arranged in a microcavity or double juxtaposed cantilever configuration. These electromagnetic forces become evident when the photodyne starts to vibrate. The oscillatory movement has been experimentally recorded and modeled theoretically. For each of the wave signals used, the frequency that optimizes the displacement has been found. As it can be seen in Figure 7, for the external frequency value of 5 Hz, the photodyne displacement achieved by using the sinusoidal signal is 2.75% greater than the triangular signal and 11% greater than the rectangular signal. For the external frequency of 10 Hz, the best option was a sinusoidal waveform which gave 16% higher displacement than the rectangular signal and 18% higher than the triangular signal. Nonetheless, for an external frequency of 15 Hz, the best option was the rectangular form, in which case the displacement amplitudes of the sinusoidal and triangular forms were 15% lower. The interesting results shown in Figure 4, Figure 5 and Figure 6 are the harmonic results. Some have large harmonic output and some have very small harmonic results. The mean reason for this is the duty cycle variation. As it increases, the number of mechanical modes is also incremented. If this parameter is not properly controlled, it may induce that the number of harmonic varies as well. The damping of the oscillations also influences how high the harmonic amplitude is going to be. Experimentally the damping is not easy to control because it depends on many nonlinearities such as the initial cavity size, surface uniformity and even the nature of the input waveform. 

The highest amplitude displacement correlates with the highest theoretical duty cycle parameter value for the three frequencies and all waveforms, (0.5, 0.5 and 0.49 for the external frequencies of 5 Hz, 10 Hz, and 15 Hz, respectively). This could be because, the longer the sample is illuminated, the more modes have the opportunity to couple as the dynamic cavity moves. In addition, the maximum mechanical power has the same correlation as the duty cycle parameter against the external frequencies and waveforms (see Figure 8). This result could be taken as a starting point to make a more exhaustive analysis in the future. The data obtained can be implemented in the experimental design and thus optimize the oscillations of the photodyne. 

The largest difference between the amplitudes of the oscillation obtained theoretically and experimentally is 2.83%. Taking into account that the theoretical model has been optimized, not only to reproduce the amplitude of the movement, but also the harmonics of each signal, it is a good fit between both results. Another important parameter is the acceleration of the photodyne movement. The values found for the largest displacements were $0.02\;m/s^{2}$ $0.05\;m/s^{2}$ and $0.09\;m/s^{2}$ for the external frequencies 5 Hz, 10 Hz and 15 Hz, respectively. These values of the acceleration are the highest obtained in the experiment for each frequency. Finally, once this type of new photonic structure is optimized, we can think of integrating them in more complicated devices such as a sensor. This is because the photodynes are based on porous silicon and some gas or liquid can be introduced inside the pores that would change the refractive indices, thereby modifying the electromagnetic force that can be generated. Thus, when the photodyne is operating, the vibrometer would detect a change in the oscillation amplitude according to the properties of the inserted material. Additionally, in the future, we would like to further explore the dangling defect condition on the force generation; that is, the impact of the inhomogeneous distribution of the air gap between both Psi multilayer foils on the creation of electromagnetic forces. We would like to determine the relationship between pumping light shapes from the chopper and charge displacement relaxation times within the multilayers.

Another important point to discuss is that, at the optical frequencies of this work, the external field should be able to excite phonon modes whereby Guided acoustic wave Brillouin scattering (GAWBS) could raise. These GAWBs should be naturally related to the mechanical effects reported in this paper. Nonetheless, for the transversal dimension of a photodyne (approximately 2 mm), the transversal sound speed of silicon (5800 m/s), the air sound speed (340 m/s) and the maximum porosity of the multilayers (88%), we can estimate GAWBS mechanical frequencies. Porous silicon sound speed would be 995.2 m/s (340 × 0.88 + 5800 × 0.12), thus 995.2/2 × 10^−3^ equals 497,600 Hz, almost half a MHz. These range of frequencies are in the correct range found elsewhere in photonic crystals [17,18] and they could be 0.5 × 10^6^ faster that the frequencies we found in our experiments. Moreover, since GAWBS is a thermal effect [18], in the past, we have done some control experiments to rule out heating effects from laser illumination. To measure the influence of laser fluctuations and temperature changes on the possible generation of mechanical oscillations in our device, a laser illuminated one photodyne device for five minutes and the vibrometer measured any possible oscillation. The result only showed a 1/*f^a^* noise signal with no peaks within the frequency band of interest (1 Hz–64 Hz), thus if GAWBS were presented they could certainly produce some mechanical oscillations but clearly it was not the case [15]. Finally, it is known that GAWBS could modulate the existence of localized photonic states [17] and, since the electromagnetic force is always maximized when a localized state is excited, a photodyne with no localized states could not maximize the electromagnetic force. Nevertheless, theoretical calculations showed that the electromagnetic force is still high (500 times higher than any current optical tweezer) even in the case where there are no localized states. Consequently, from all these arguments, GAWBS cannot explain the nature of the oscillations we have found in this work and in the past [13,14,15] but they may contribute as noise within our measurements. For future work, experimental studies on measuring GAWBS could be included. This can be interesting for sensor applications since the GAWBS peaks are highly sensitive to strain and temperature. 

Finally, in every oscillator, it is essential to estimate the phase noise within the oscillation output signal. The easiest way to do it is using an oscillation signal that only contains a single frequency. In the past, we have created self-oscillators (where there is only one oscillation frequency) using exactly the same configuration and experimental setup. Phase noise can be estimated using the power spectral density of the oscillation signal (frequency domain) and the temporal jitter of the same signal (temporal domain). If we use the frequency domain, the phase noise is found as the ratio of the noise power in a 1 Hz bandwidth and the total signal power. Using the power spectral density of a self-oscillation experiment (see Supplementary Materials), the phase noise was estimated with a value of −7.17 dB/Hz @ 20.8 Hz. In the time domain, we used 20 successive measurements of the period of the self-oscillator (see Supplementary Materials). If the measurements contain only random jitter elements, the distribution of these elements would be a Gaussian and the RMS jitter would be given by the value of one standard deviation. Then, the phase noise can be approximated by $20Log_{10}\left(\sigma \omega /2\right)/\Delta f$ [19], where ω is the oscillation frequency, σ is the RMS jitter and Δf is the frequency interval for the full width at half maximum, which can be obtained from the power spectral density. Bear in mind that this phase noise approximation is valid only if its value is almost constant within such frequency interval. We used a one-sample Kolmogorov–Smirnov test to verify that a Gaussian distribution described the jitter elements. The test was passed with a probability of 94%. We found that σ=0.011 s, ω=2π(20.8) rad/s and Δf=2.66 Hz. Using these values, the phase noise is −8.51 dB/Hz @ 20.8 Hz, which is of the same order of magnitude as the value found in the frequency domain. We do not expect this value to be that different from our current experiments, but, in the future, we would like to measure the phase noise more precisely and determine the different sources of noise by means of a spectrum analyzer and with better experimental controls. 

## Supplementary Materials

The following are available online at http://www.mdpi.com/1996-1944/11/5/854/s1.
