# Supplementary figures and images for: The Influence of the External Signal Modulation Waveform and Frequency on the Performance of a Photonic Forced Oscillator
Source: Materials (Basel). 2018 May 21;11(5):854. doi: 10.3390/ma11050854 (PMC5978231; doi:10.3390/ma11050854)

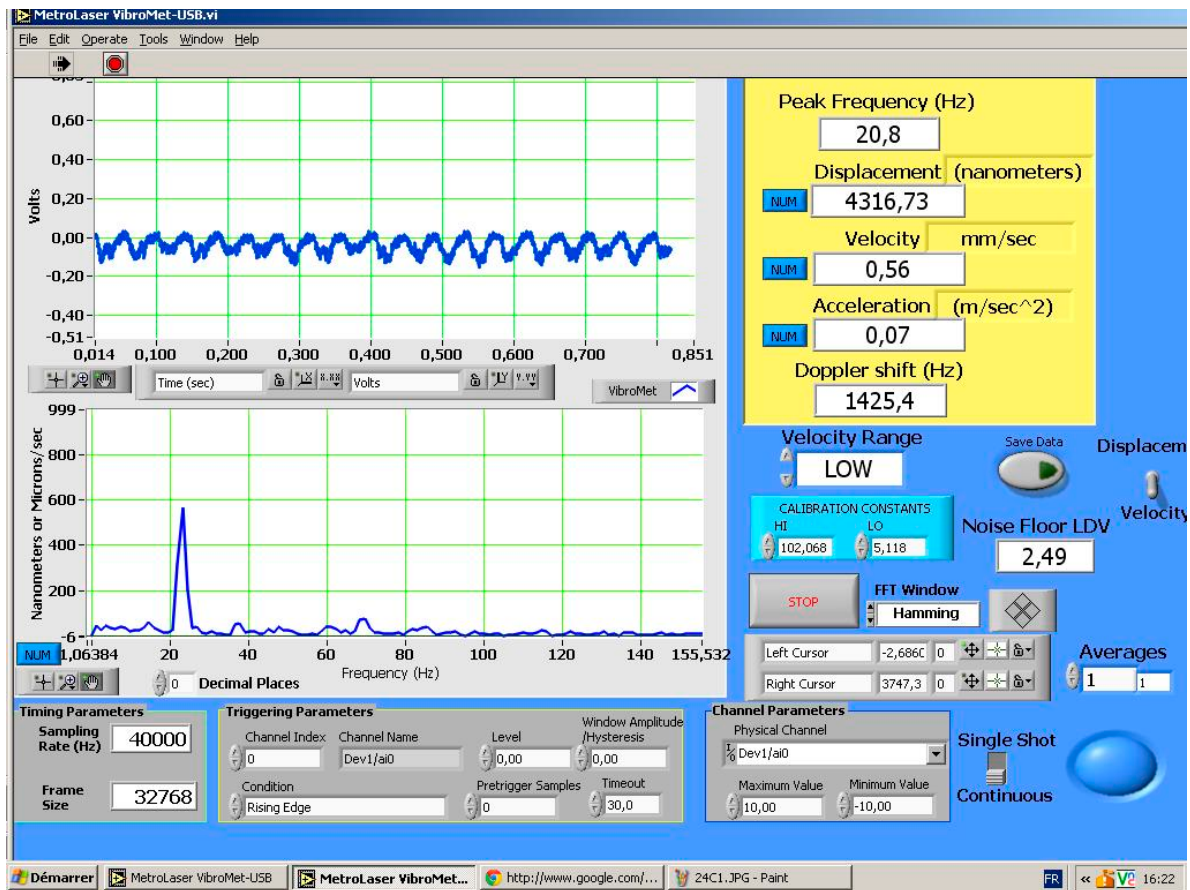

Supplement: Supplementary file 1 [file materials-11-00854-s001.pdf]
